# Antioxidant potential of the diet in Italian children with food allergies

**DOI:** 10.3389/fnut.2023.1096288

**Published:** 2023-03-16

**Authors:** Erica Pendezza, Chiara Nava, Alessandro Leone, Francesca Riccaboni, Alessandra Bosetti, Gian Vincenzo Zuccotti, Simona Bertoli, Enza D’Auria

**Affiliations:** ^1^Department of Pediatrics, Buzzi Children’s Hospital, Milan, Italy; ^2^Department of Food, Environmental and Nutritional Sciences (DeFENS), University of Milan, Milan, Italy; ^3^Department of Biomedical and Clinical Sciences, Buzzi Children’s Hospital, University of Milan, Milan, Italy; ^4^Obesity Unit and Laboratory of Nutrition and Obesity Research, Department of Endocrine and Metabolic Diseases, IRCCS Istituto Auxologico Italiano, Milan, Italy

**Keywords:** food allergies, dietary antioxidants, ORAC, antioxidant potential, children

## Abstract

A reduced fruit and vegetable consumption, which implies a decreased intake of antioxidant compounds, seems to play a role in allergic diseases onset. Data on the antioxidant capacity of diet in children with food allergies, who are on an avoidance diet, are still lacking. This pilot study aims to assess the antioxidant potential of diet in Italian children with food allergies, compared to healthy children, using the oxygen radical absorbance capacity (ORAC) method. 95 children (54 with confirmed food allergies and 41 controls), with a median age of 7.8 years, were enrolled and underwent a nutritional assessment. Mean nutrient intakes were compared using the Mann–Whitney test. ORAC resulted significantly lower in allergic children (median 2,908, IQR: 1450;4,716) compared to control children (median 4,392, IQR: 2523;5,836; *p* = 0.049). Among micronutrients with antioxidant properties, vitamin A intakes were significantly higher in controls than in allergic children. Using Spearman’s correlation, a moderate-to-strong correlation between ORAC and vitamin C, potassium and magnesium was observed (*ρ* = 0.648, *p* < 0.001; *ρ* = 0.645, *p* < 0.001; *ρ* = 0.500, *p* < 0.001, respectively). Iron, phosphorus, vitamin E and vitamin A intakes were also moderately-to-low correlated with ORAC values (*ρ* = 0.351, *p* < 0.001; *ρ* = 0.367, *p* < 0.001; *ρ* = 0.346, *p* < 0.001; and *ρ* = 0.295, *p* = 0.004, respectively). We hypothesize that the reduced antioxidant potential of the diet might be related to a reduced variety of the diet in children with food allergies. Our study suggests that the diet of children with food allergies has a lower antioxidant potential (expressed as ORAC value) compared to the diet of healthy children, regardless of the allergenic food excluded from the diet. This issue should be further investigated in prospective, powered studies.

## 1. Introduction

Over the past decades, the burden of food allergies has significantly increased, with an estimated prevalence in European school-aged children currently ranging between 1.4 and 3.8% (1). There is some evidence that food allergies in infancy may progress to rhinitis and asthma over time; these occurrences are referred to as the ‘atopic march’ (2, 3). As the pathogenesis of allergic diseases appears to be so complex, and far from fully understood, multiple factors have been proposed as risk factors; among them, diet, meaning that both nutrient and non-nutrient factors have been called into question as possible causes in the development of food allergies and other allergic diseases (4, 5).

Since systemic inflammation and oxidative stress are mechanisms involved in the pathogenesis of allergic diseases (6–8), it has been hypothesized that dietary antioxidant factors may play a role in their onset and epidemiological studies aiming to investigate this association have been conducted (6, 9, 10).

Lower intakes of antioxidants have been demonstrated to increase the risk of allergic diseases (11–15).

Of note, many of the trials investigating the association between antioxidants and allergic diseases until now have focused on dietary intake or plasma levels of a single nutrient, such as specific vitamins and/or micronutrients, rather than a cumulative antioxidant dietary measurement (15). However, there is an emerging understanding that because nutrients and foods are not eaten in isolation, the antioxidant effect of foods is likely attributable to a variety of dietary compounds, which operate in a synergistic way (16).

Also noteworthy is that an inverse association between fruit and vegetable consumption with a high adherence to a traditional Mediterranean diet during childhood, and concurrent symptoms of asthma, has been observed in Greece (17, 18).

This may be explained by the fact that the Mediterranean diet, which is traditionally high in fruit, vegetables, dietary fiber and omega-3 fatty acids, has been linked to anti-inflammatory pathways (19).

Beyond the measurement of individual nutrients with a known antioxidant activity, the total antioxidant capacity of the diet becomes more valuable and more challenging to assess. With an increasing knowledge of and interest in antioxidants, several methods for measuring the antioxidant potential of foods have been developed (20).

The oxygen radical absorbance capacity (ORAC) method assesses the ability of a fluorescent antioxidant contained in food extracts to inhibit the peroxyl radical-induced degradation (21).

Oxygen radical absorbance capacity values are expressed as μmol of TE/gr (or μmol of TE/L), where TE stands for Trolox equivalents (Trolox is a synthetic molecule, water-soluble analogue of Vitamin E) (21).

In order to reflect the solubility of all measurable antioxidants, two variants of the ORAC value have been proposed: hydrophilic ORAC (H-ORAC) and lipophilic ORAC (L-ORAC).

The former is used for water-soluble antioxidants such as vitamin C and most polyphenols, while the latter is suitable for fat-soluble compounds such as vitamin E and polyphenol esters (20).

The combination of H- and L-ORAC has allowed a set of ORAC values for a large number of foods to be obtained (22, 23).

Among foods with high ORAC are those rich in compounds with antioxidant properties, such as vitamin A, vitamin C, carotenoids, tocopherols and polyphenols. These include fresh fruit, yellow-orange-red vegetables, berries and some nuts (23).

The study by Gref et al. (24) examined the nutrient intakes in a cohort of over 2,350 children, by using the ORAC value as an indicator of the total antioxidant capacity of the diet. Authors found a significant inverse association between total antioxidant capacity of the diet and the incidence of sensitization to environmental inhalants.

To our best knowledge, data on the antioxidant capacity of diet in children with food allergies, who are on an avoidance diet, are lacking.

This study aims to assess the antioxidant potential of the diet in children with food allergies, by using the ORAC value, and to compare it to healthy children. In addition, we explore the correlation between the intake of some specific micronutrients with antioxidant properties and ORAC values.

## 2. Methods

### 2.1. Study design

This study is part of a study aimed at evaluating nutrient intake in Italian school-aged children with food allergies. This was a non-randomized case–control study comparing children with food allergies to healthy children (control group) (25).

Children with confirmed food allergies were recruited from children who had been referred to the Vittore Buzzi Children’s Hospital Allergy Unit (Milan, Italy) between May 2017 and December 2019. All children with a suspected food allergy underwent a complete diagnostic work-up including a confirmatory oral food challenge, except for children with a history of anaphylaxis. Only children with a confirmed diagnosis of an allergy to one or more foods (both IgE or non-IgE-mediated) were enrolled in the study.

A control group was recruited from healthy children who had been referred for nutritional status assessment to the International Centre for the Assessment of Nutritional Status (ICANS) at the University of Milan.

Exclusion criteria were the presence of chronic (e.g., coeliac disease) or acute (e.g., influenza) diseases, a restricted diet (e.g., a vegetarian/vegan diet, exclusion of some food groups from the diet) and any pathological conditions with dietary implications.

All children underwent a nutritional assessment, during which their parents were asked to fill out a three-day food dietary record.

A total of 95 children (54 with food allergies and 41 controls), with a median age of 7.8 years, were enrolled.

The study was performed in accordance with the Declaration of Helsinki and the parents of each child gave their written and informed consent. The study protocol was approved by the Ethics Committee of Milan Area 1 (2018/ST/267).

### 2.2. Nutrient intake

A prospective 3-day food dietary record, including two non-consecutive working days and one weekend day, was used to estimate nutrient intakes.

Parents were trained by an expert dietitian on how to fill out the dietary record, reporting in detail on every food item or beverage consumed during the day, the time of consumption and the amount consumed (weighed before cooking whenever possible).

Parents were also instructed to weigh and record any leftovers and to quantify food or beverages using common kitchen utensils, such as glasses, spoons or bowls, to estimate portions whenever weighing was not possible. When children ate in the school canteen, parents were asked to provide the school menu with portion weights, and the portion consumed by each child was estimated according to the teachers’ directions.

Nutrient intakes were estimated from the three-day dietary record by using the Metadieta software (Me.Te.Da. S.r.l., San Benedetto Del Tronto, Italy). With regard to the ORAC index, the software uses the data provided by the USDA Foods Table, with the ORAC index of selected foods expressed as μmol of Trolox Equivalents per 100 g of foods (μmol TE/100 g). Values were compared to the recommended intake for sex and age according to the Italian Reference Nutrient Intakes (RNI; Società Italiana di Nutrizione Umana 2014) (26).

### 2.3. Statistical analysis

Continuous variables are reported as median and interquartile range, as many of the variables did not follow a normal distribution. Discrete variables are reported as count and percentage. Nutrient intakes were adjusted for energy intake using the residual method (27).

Negative consumption values resulting from this adjustment were set to 0 for interpretability. The mean nutrient intakes of allergic and non-allergic children were compared using the Mann–Whitney test. In order to understand which nutrients contributed to increasing ORAC, nutrient intakes were correlated with ORAC values using Spearman’s correlation. The magnitude of the correlation was considered weak if *ρ* (rho) was < 0.3, low if it was between 0.3 and 0.5, moderate if it was between 0.5 and 0.7, and strong for *p* values > 0.7. A value of *p* of < 0.05 was considered statistically significant. Statistical analysis was performed using STATA 12.0 software.

## 3. Results

A total of 95 children (54 with food allergies and 41 controls) with a median age of 7.8 years (IQR: 6.0; 10.7) were recruited for the study. The two groups were age-comparable.

Of the 54 children with food allergies, 15 were allergic to a single food, 7 to two foods and 32 to three or more foods. The age, sex and allergy distribution for the food allergy and control groups are shown in Table 1.

**Table 1:** Age, sex and allergy distribution of children with and without food allergies.

	Total (*n* = 95)	Control (*n* = 41)	Allergic (*n* = 54)
Age (median, years)	7.8	8.3	7.5
Gender (M)	*n* = 49 (51.6%)	*n* = 19 (46.3%)	*n* = 30 (55.5%)
Single food allergy			*n* = 15 (27.7%)
Two-foods allergy			*n* = 7 (13%)
Multiple food allergy			*n* = 32 (59.3%)

The foods most commonly responsible for food allergies in our sample were tree nuts (*n* = 35), cow’s milk (*n* = 14) and hen’s egg (*n* = 12). Other foods responsible for allergic symptoms were wheat, fish, shellfish, mollusks, legumes, spelt, oats, barley, sesame, vegetables, millet and beef.

The energy and micronutrient intakes and ORAC values of the two groups of children, as estimated from the dietary records, are shown in Table 2.

**Table 2:** Energy and micronutrient intakes of children with and without food allergies.

	Total (*n* = 95)			Control (*n* = 41)			Allergic (*n* = 54)			*p* Value
	Median	P25	P75	Median	P25	P75	Median	P25	P75	
Age (years)	7.8	6.0	10.7	8.3	6.6	10.2	7.5	6.0	10.9	0.543
Energy (kcal/day)	1,570	1,335	1948	1,558	1,341	1820	1,668	1,332	2000	0.550
Ca	433.7	278.3	572.1	503.6	338.1	632.9	355.6	202.7	545.7	0.003^*^
Na	1257.5	901.1	1683.8	1375.0	1014.8	1720.2	1181.7	850.1	1651.1	0.179
K	1710.3	1374.7	2060.1	1655.6	1490.1	2108.2	1779.2	1267.9	2040.5	0.439
P	727.2	560.7	876.2	766.1	652.8	861.7	703.1	528.2	876.9	0.151
Fe	6.2	5.2	7.5	6.0	4.8	6.6	6.5	5.4	8.1	0.067
Zn	6.2	4.9	8.1	6.5	5.5	8.3	6.1	4.3	7.9	0.036^*^
Mg	121.5	96.2	145.4	123.0	102.5	142.7	120.8	92.2	146.1	0.707
Vit. A	693.6	441.7	1081.6	746.6	634.3	1177.1	618.7	340.6	891.5	0.019^*^
Vit. C	54.9	28.4	93.5	56.5	26.8	89.9	53.6	31.3	113.2	0.833
Vit. D	1.0	0.7	1.9	0.9	0.7	1.4	1.3	0.6	2.4	0.398
Vit. E	7.1	5.9	8.3	6.9	5.8	8.1	7.3	5.9	8.3	0.568
Vit. B12	2.4	1.5	3.1	2.4	1.6	3.0	2.3	1.4	3.3	0.781
ORAC	3248.42	2073.78	5619.47	4392.50	2522.96	5836.49	2908.05	1450.41	4716.44	0.049^*^

Values are median and interquartile range (P25–P75). Nutrient intakes are adjusted for energy intake using the residual method. ^*^p < 0.05

Oxygen radical absorbance capacity was found to be significantly lower in allergic children (median: 2908, IQR: 1450; 4,716) than in control children (median: 4392, IQR: 2523; 5,836; *p* = 0.049). Among micronutrients with antioxidant properties, vitamin A intakes were found to be significantly higher in control children than in allergic children.

We also examined the nutrients that contributed to ORAC.

Table 3 shows the correlation coefficients between nutrient intakes and ORAC values. We found vitamin C, potassium and magnesium to be moderately-to-strongly correlated with ORAC (0.648, 0.645, 0.500, respectively), followed by iron, phosphorus and vitamin E, whose intakes were also correlated with ORAC (0.351, 0.367, 0.346, respectively). Finally, vitamin A intake showed a weak correlation with ORAC values (0.295).

**Table 3:** Spearman’s rank correlation coefficients.

Variables	ORAC	Ca	Fe	P	Na	K	Mg	Vit. E	Vit. C	Vit. A	Vit. D	Vit. B12
ORAC	1.000											
Ca	0.152	1.000										
Fe	0.351^*^	0.161	1.000									
P	0.367^*^	0.650^*^	0.378^*^	1.000								
Na	–0.032	0.011	0.028	–0.035	1.000							
K	0.645^*^	0.242^*^	0.635^*^	0.500^*^	–0.087	1.000						
Mg	0.500^*^	0.389^*^	0.642^*^	0.670^*^	–0.247^*^	0.740^*^	1.000					
Vit. E	0.346^*^	0.053	0.409^*^	0.279^*^	–0.077	0.278^*^	0.409^*^	1.000				
Vit. C	0.648^*^	–0.049	0.396^*^	0.278^*^	–0.167	0.668^*^	0.535^*^	0.481^*^	1.000			
Vit. A	0.295^*^	0.239^*^	0.252^*^	0.186	0.133	0.312^*^	0.239^*^	0.327^*^	0.197	1.000		
Vit. D	0.066	–0.087	0.303^*^	0.210^*^	0.022	0.207^*^	0.144	0.287^*^	0.168	–0.102	1.000	
Vit. B12	0.132	0.315^*^	0.438^*^	0.569^*^	–0.043	0.390^*^	0.390^*^	0.238^*^	0.204^*^	0.091	0.425^*^	1.000

* *p* < 0.05.

## 4. Discussion

To our best knowledge, this is the first study aiming to specifically investigate the overall antioxidant diet capacity in children with food allergies, compared to healthy children.

Our findings show that the antioxidant potential of the diet, expressed through ORAC value, is significantly lower in allergic children than in healthy children, regardless of the food allergen involved. This finding is novel and quite unexpected, as most of our children were allergic to cow’s milk and/or eggs, in accordance with the prevalence of these allergies in Europe (1), and not to foods with a higher ORAC value, such as fruit or vegetables. This result may suggest that children in our sample consume a reduced variety of foods in their diet, which also extends to foods with a high ORAC value.

Of interest, similar results have been observed in a trial that investigated dietary habits and antioxidant potential of the diet in children suffering from Crohn’s disease (not following dietotherapy) and which found ORAC to be lower than in healthy children (28).

In addition, the ORAC value has been studied in relation to an adherence to the Mediterranean Diet in a group of healthy adolescents from the DIMENU (DietaMediterranea and Nuoto) study (29).

Authors found that, even with a similar total energy intake, adolescents with a higher adherence to the Mediterranean Diet had higher ORAC values compared to those with a poorer adherence.

This finding is not surprising, considering that many foods that are the basis of the Mediterranean diet are characterized by a high ORAC value.

We can observe that the median ORAC value in our healthy children (4392.50 μmol TE) is quite close to the observed medium value of the DIMENU study (4196.97 μmol TE).

In our study, nutrient intakes were adjusted for energy intake, which means that their intake was observed independently of total dietary energy intake. Not taking this adjustment into account could have represented a confounding factor.

As concerns vitamin A, we found a reduced intake in allergic children, compared to healthy children. This finding could be explained by the fact that vitamin A, in the form of retinol, is mainly found in milk and dairy products (butter, fresh and aged cheeses, yogurt) and eggs, and especially in egg yolk (30).

Previous trials have also reported a significantly reduced vitamin A intake in children who have a cow’s milk allergy and follow an avoidance diet (31, 32).

A significant correlation between the ORAC value and the intake of some micronutrients has also been observed, the strongest one being between ORAC and vitamin C, and between ORAC and potassium.

The correlation with vitamin C is not surprising, as Vitamin C, also known as ascorbic acid, is one of the essential trace elements in the human body and considered to be the most effective water-soluble antioxidant (33, 34).

Conversely, the correlation between ORAC value and potassium appears less predictable. We can speculate that it might be secondary to the fact that vitamin C is present in abundance in fruit and vegetables, foods equally rich in potassium (30).

We also found a moderate correlation between ORAC and vitamin E, lower than that observed for ORAC and vitamin C. This result is in accordance with the actual antioxidant activity which has been demonstrated to be higher for vitamin C than for vitamin E (35).

A low-to-moderate correlation was also found between ORAC and magnesium. Magnesium antioxidant activity is recognized, as its deficiency has been related to higher levels of oxidative stress markers and a weakened antioxidant defense (36, 37).

Thus, similarly to potassium, this correlation could be explained by the presence of magnesium in foods equally rich in other antioxidants, such as tree nuts which are also a good source of vitamin E, or some vegetables also rich in vitamin C (30).

Of note, we observed a reduced antioxidant potential of the diet also in children who were not allergic to fruit and vegetables. In our sample, the foods most commonly responsible for food allergies were tree nuts, milk and eggs, while only 13 children were allergic to fruit and/or vegetables.

We hypothesize that the reduced antioxidant potential of the diet might be related to a reduced variety of the diet itself, intended as a reduced number of different food or food groups consumed over a specific reference period (38, 39).

Actually, in children with food allergies, several factors have been reported which may negatively influence diet diversity. For instance, parents or caregivers may be afraid of triggering adverse reactions by introducing new foods; moreover, children themselves may manifest neophobia and/or tendency to reject foods they have never tried and which they see as a potential threat (40, 41). On the one hand, limiting food choice to a range of “familiar” known foods may help to ensure a greater sense of safety and confidence for the child and caregivers.

However, if this reduced dietary variety persists over time, it could place children with food allergies at a greater risk of nutritional inadequacies, especially micronutrient deficiencies.

The main limitation of our study is due to its small sample size, which did not allow us to stratify by age group or by allergen. In addition, the trial was not randomized.

Conversely, the strength and the novelty of our study is in its assessment of the overall antioxidant potential of the diet, by using the ORAC index, in a sample of children with food allergies who are on an avoidance diet. Further powered studies are warranted to confirm these findings.

## 5. Conclusion

The present study suggests that the diet of children with food allergies has a lower antioxidant potential diet (expressed as ORAC value) compared to the diet of healthy children, regardless of the allergenic food excluded from the diet.

Our results highlight the importance of tailored nutritional counseling for children with food allergies, who need to exclude certain food groups from their diet.

The antioxidant potential of the diet deserves to be further investigated in powered-prospective studies in order to test whether reduced antioxidant capacity may influence the course of the disease and also to assess whether an increased intake of dietary antioxidants could result in benefits for the treatment of allergies.

## Data availability statement

The original contributions presented in the study are included in the article/supplementary material, further inquiries can be directed to the corresponding author.

## Ethics statement

The studies involving human participants were reviewed and approved by Ethics Committee of Milan Area 1 (2018/ST/267). Written informed consent to participate in this study was provided by the participants’ legal guardian/next of kin.

## Author contributions

ED’A: conceptualization. EP and AB: data collection. AL: data analysis and interpretation. EP, CN, and FR: writing—original draft. EP, AL, and ED’A: review and editing. GZ and SB: supervision. All authors contributed to the article and approved the submitted version.

## Conflict of interest

The authors declare that the research was conducted in the absence of any commercial or financial relationships that could be construed as a potential conflict of interest.

## Publisher’s note

All claims expressed in this article are solely those of the authors and do not necessarily represent those of their affiliated organizations, or those of the publisher, the editors and the reviewers. Any product that may be evaluated in this article, or claim that may be made by its manufacturer, is not guaranteed or endorsed by the publisher.
